# Tire Condition Monitoring Using Transfer Learning-Based Deep Neural Network Approach

**DOI:** 10.3390/s23042177

**Published:** 2023-02-15

**Authors:** Vinod Vasan, Naveen Venkatesh Sridharan, Anoop Prabhakaranpillai Sreelatha, Sugumaran Vaithiyanathan

**Affiliations:** School of Mechanical Engineering (SMEC), Vellore Institute of Technology, Chennai 600127, India

**Keywords:** tire condition monitoring system, machine learning, deep learning, pretrained networks, radar plots, image classification

## Abstract

Monitoring tire condition plays a deterministic role in the overall safety and economy of an automobile. The tire condition monitoring system (TCMS) alerts the driver of the vehicle if the inflation pressure of a particular tire decreases below a specific value. Owing to the high costs involved in realizing this system, most vehicles do not feature this technology as a standard. With highly robust and accurate sensors making their way into an increasing number of applications, obtaining signals of varied types (especially vibration signals) is becoming easier and more modularized. In addition, feature-based machine learning techniques that enable accurate responses to varied input conditions have sought greater scientific attention. However, deep learning is gradually finding greater applications pertaining to condition monitoring. One approach of deep learning is presented in this paper, which instantaneously monitors the vehicle tire condition. For this purpose, vibration signals were obtained through the rotation of the tire under different inflation pressure conditions using a low-cost microelectromechanical system (MEMS) accelerometer.

## 1. Introduction

Transportation technologies and channels are global economic arteries that maintain a supply–demand balance of consumer goods, bridge geographical barriers to support point-to-point movements and accelerate infrastructural progress to improve quality of living. The essence of locomotion lies in the ability of a vehicle to move. The development of pneumatic tires was a revolutionary step forward in vehicular mobility. Constant improvements in tire thread design, weight reduction and manufacturing processes have resulted in higher consumer demand and, therefore, increased dividends for tire manufacturers. Fuel efficiency, ride quality and, most importantly, occupant safety are important factors to be considered from two perspectives—a designer point-of-view and a consumer perspective. Tires play a crucial role in guaranteeing driver and passenger safety under adverse driving conditions. Therefore, priority must be given to robust tire design and manufacturing. In addition, maintenance of the tire is equally critical to ensure long life of usage, sustained fuel economy and persistent occupant safety in the vehicle.

Vehicle tires can run for thousands of kilometers before requiring replacement. However, improper pressurizing of tires below or beyond rated pressures and heavy-footed driving can accelerate wear and tear, drastically reducing product lifetime. This affects fuel economy through increased rolling resistance. This, in turn, leads to a cascade of consequences, especially inducing a negative impact on the environment due to more frequent refueling and premature disposal of tire sets, which accumulate and stagnate. Tires pose a significant hazard to the environment due to the non-biodegradability of their base materials and chemical additives. With the high initial cost for a set of fresh automobile tires and fuel costs on the rise, consumers tend to prefer options with appropriate quality and cost blend when in the market for new or used automobiles and auto components. These factors evidently substantiate the need to enhance the longevity of tires by monitoring the current tire condition for preventive and predictive maintenance through tire pressure monitoring systems (TPMSs). The currently available TPMSs include:

1. Indirect TPMS (iTPMS)—These systems rely on pre-existent sensors as part of the electronic stability control (ESC) and antilock braking systems (ABSs) of the vehicle and advanced spectral analysis techniques, which relay tire pressure level based on current wheel diameter and wheel angular velocity.
2. Direct TPMS—Direct TPMS utilizes exclusive hardware, such as pressure sensors mounted on the tire-valve stem or wheel hub of vehicles, which then convey pressure information to the computer control unit of the vehicle.

Development in the field of TPMS is gradually gaining momentum. In 2017, Silalahi et al. examined the design of a TPMS using a pressure sensor, microcontroller and Bluetooth communication protocol [1]. This was based on the original approach adopted by Hasan et al., encompassing a pressure sensor, signal conditioning unit, switch, RF transmitter microcontroller and long-life battery [2]. In 2021, Lee et al. investigated the development and implementation of an indirect TPMS based on adaptive extended Kalman filtering of vehicle suspension systems [3]. Traditional machine learning requires explicit feature selection and extraction processes to be performed before feature classification is carried out. This proves to be a complicated task to handle with the increase in complexity of the features to be recognized and classified. On the other hand, deep neural network (DNN) learning strategies enable the machine to learn without explicitly extracting features from image formats of data plots corresponding to mappable features. With the availability of specialized sensors and computer systems that possess signal capture, sampling and augmentation techniques, raw input data can be extracted from the experimental setup with relative ease.

**Figure 1:**
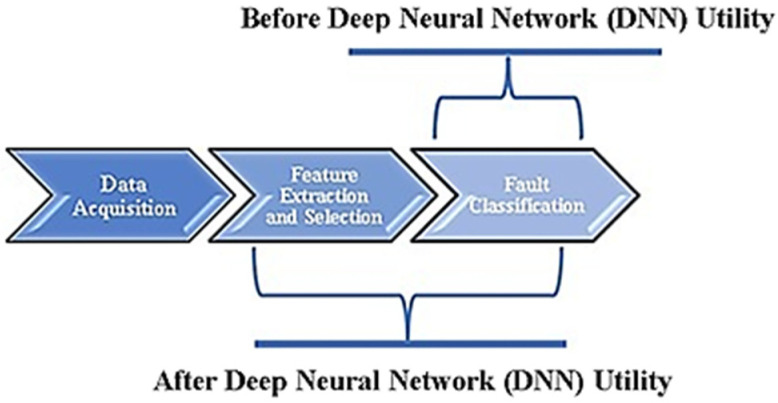
Application of deep learning before and after DNN utility.

The deep neural network (DNN) strategy encompasses hierarchically stacked neural layers that obtain input information from the user. With the raw data as input, the deep learning network understands the structural makeup of a complex data set, enabling it to ‘learn’ the most significant set of features through several layers. The term ‘deep’ is used to suggest that the information received as input in the form of raw data is not just passed on to a single successive layer for further processing but is rather allowed to percolate through numerous levels of layered procedures. In effect, deep learning has the potential to present disruptive benefits over traditional machine learning and statistical approaches in fault diagnosis and condition monitoring. The application of DNN in the condition monitoring and fault diagnosis domain has remained in its premature stages for a considerable time. However, increasing efforts are being made to deploy neural network advantages to resolve a wider variety of challenges. A deep statistical feature learning approach for diagnosing bearing and gearbox conditions based on statistical features extracted in the frequency, time and time-frequency domains was proposed by Li et al. [4]. On the other hand, Shao et al. performed roller-bearing fault detection through an optimized deep belief network through 18 time domain features [5]. A convolutional neural network (CNN) was proposed by Chen et al. to diagnose gearbox condition using signals extracted in the time and frequency domain [6]. In these instances, deep learning networks were used as conventional alternatives to traditional classifiers, and the complete potential of a DNN was untapped (as depicted in Figure 1) until at least 2015. With progressively demanding condition monitoring situations, the feature learning and selection simplicity of neural networks provided optimal choices for the computerized classification of fault conditions within short time intervals. Some works related to the use of deep learning methods in mechanical systems are presented in Table 1.

Roller bearing condition monitoring using DNN with two branches—one branch accounting for feature engineering (pattern recognition and extraction) and the other branch taking care of the final fault classification [15]—was performed by Guo et al. The convolutional long short-term memory (C-LSTM) approach was employed by Zhao et al. to monitor the condition of a tool [16]. Xiao et al. performed a study into the adoption of a novel joint transfer network for unsupervised bearing fault diagnosis from the simulation domain to the experimental domain [17]. Cai et al. examined a data-driven methodology for fault diagnosis in a permanent magnet synchronous motor using Bayesian networks [18]. The intelligence and condensed characteristics of DNN through its feature learning capability have earned this network growing recognition and adoption as a largely automated methodology (as depicted by Figure 1). The use of pretrained networks helps researchers focus on the core problem to be solved using artificial neural networks rather than the programmability of the network itself. Pretrained neural networks have found applications in the field of medicine, more specifically, in ophthalmology for diabetic retinopathy detection, as examined by Mateen et al. [19]. Nogay et al. researched the detection of epileptic seizures using a deep learning CNN (pretrained) advocating for transfer learning [20]. Kumari et al. examined the application of a pretrained CNN in forensics for offline signature detection [21], while Shibli et al. investigated the implementation of pretrained CNN for artificial intelligent drone-based encrypted machine learning of image extraction [22]. In 2021, Rajadurai et al. examined the detection of cracks in concrete surfaces through deep learning vision using AlexNet CNN [23], and Sharma et al. evaluated the identification of vehicles using region-based CNN with an intelligent transportation focus [24].

Convolution neural networks (CNN) can learn complex features from the input image information and, as such, are regarded as robust modus operandi in the domains of fault diagnosis, natural language processing and object recognition. In the current research, four pretrained networks, namely GoogLeNet [25], VGG-16 [26], AlexNet [27] and ResNet-50 [28], were evaluated based on their ability to classify graphical counterparts of tire conditions into one of four possible condition states: normal, idle, high and puncture. These four conditions of tire pressure were classified into Normal, Idle, High and Puncture, respectively. Tire condition classification is based on the vibration signals obtained for each condition of the running tire, which is subsequently translated into radar plots, which serve as the basis for comparison and classification. Radar plots can be beneficial in cases when the comparison of data trends is to be studied, making the process of feature extraction even more intuitive. In this study, we try to verify whether DNN could effectively perform condition monitoring of air-filled pneumatic tires and present better classification performance compared to traditional feature-based statistical machine learning methods. The effectiveness of the pre-trained networks was examined as a function of their respective classification accuracies by varying hyperparameters such as batch size, train-test split ratio, initial learning rate, solver type and epochs during the learning phase of each network. The approach to the experiment is summarized below:

**Technical Contributions of this Study:**

**Figure 2:**
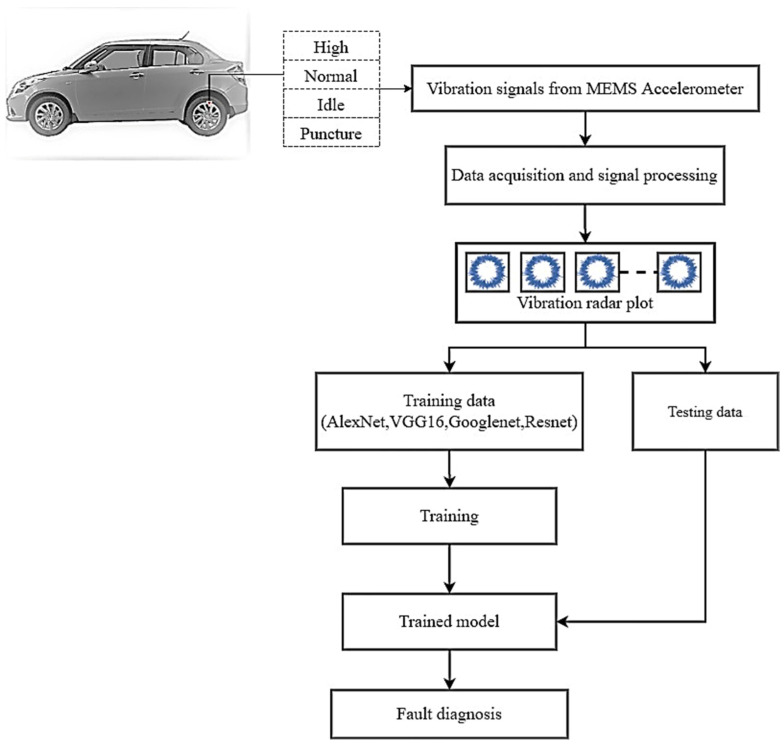
Process diagram depicting the stages involved in the current study.

- Figure 2 presents the overall process of this experimental study. Vibration data was acquired using a low-cost MEMS accelerometer for each tire condition of an air-filled pneumatic tire balanced using two 20 g weights. This vibration data was converted into radar plots and resized into sizes compatible with each of the four pretrained networks considered for this experiment—AlexNet, GoogLeNet, ResNet-50 and VGG-16—for the classification of current tire state.
- Once the radar plots corresponding to each tire condition were obtained, the plots were input to the pretrained networks under examination in image format. The hyperparameters of each of the pretrained networks were varied sequentially, namely, the training–testing split ratio value, solver algorithm, initial learning rate value and the value of batch size.
- Pretrained networks classified input images into one of four possible tire conditions—high, normal, low and puncture. After the classification results of each network were obtained, the configuration and hyperparameter values of the network exhibiting the highest accuracy under specific hyperparameter settings were tabulated, and the most optimal DNN pretrained network was presented as the best choice for implementation in tire condition monitoring using the transfer learning approach. In this experiment, it was found that ResNet-50 exhibited the highest overall classification accuracy of 93.80%, with a train–test split of 0.80, initial learning rate of 0.0001 and batch size of 10, using the RMSPROP solver.

**Novelty of the study:**

- The experimental approach presented in this paper visualizes vibration data in the form of easily interpretable radar plots in image format, generating user-friendly and flexible word processing software. This approach is in contrast to the techniques of image generation using fast Fourier transforms (FFT), Hilbert Huang transform, discrete orthonormal Stockwell transform and empirical mode decomposition methods, which require sound computer and mathematical knowledge on the part of the researcher to be executed correctly.
- The current study makes use of a low-cost MEMS accelerometer, which is readily available and produces accuracies similar to that yielded by more expensive accelerometers for large data sets. This has been achieved through the classification robustness of pretrained neural networks.
- Additionally, this experimental study involves variations of hyperparameters for each of the pretrained networks being studied to obtain the most optimal configuration for each network operating on the same data set. This facilitates the highest possible classification accuracy by each pretrained network when producing the required results.

## 2. Salient Features of the Experiment Setup

This section presents a detailed description of the experiment setup and procedure employed to perform tire condition monitoring using pretrained neural networks.

### 2.1. Experimental Setup

**Figure 3:**
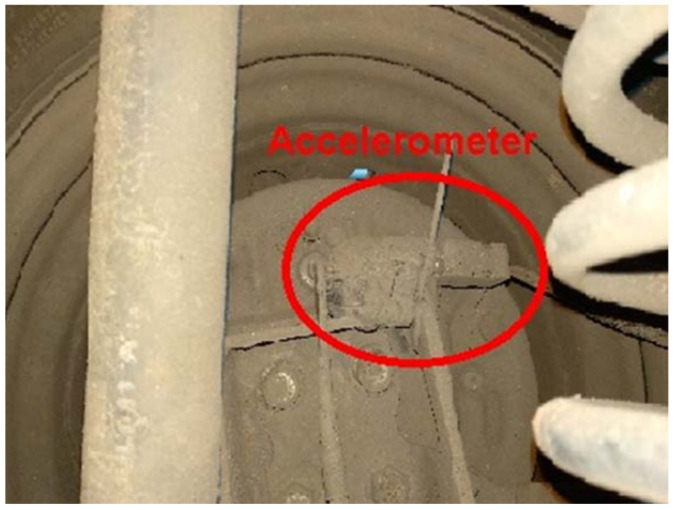
MEMS Accelerometer attached to the rear axle of the test vehicle.

For the purposes of this experiment, a vehicle with front-wheel drive was selected, and the rear left wheel axis of the vehicle’s air-filled pneumatic tire was used to extract vertical vibration signals. On the rear left wheel hub, a waterproof sealant was applied on a tri-axial MEMS accelerometer (MMA7361L with a resonant frequency of 6 kHz, frequency range of 1 to 400 Hz, and sensitivity of 206 mV/g). In order to measure vertical vibrations, this study focuses on the Y-axis of the accelerometer data. Figure 3 represents the MEMS accelerometer used for obtaining vibration data for this study.

### 2.2. Method of Data Acquisition

A data acquisition device (DAQ)—suitable to turn analog signals into digital signals and subsequent signal conditioning—was connected to the output of the accelerometer. The NI USB-6001 featuring a resolution of 14 bits, 12 input channels and a maximum sampling rate of 20 kS/s, was selected for data acquisition in this study. The NI LabVIEW software interface was used to link the DAQ output to the computer system. In principle, analog vibrations from the accelerometer were input into the DAQ system, which are subsequently converted into digital signal outputs. As an added precaution against external electronic interference, data from the accelerometer were sent to the DAQ through a shield wire. Using NI Lab VIEW software, the DAQ output was connected to the monitoring computer system.

### 2.3. Experimental Procedure

In the current experiment, under normal driving conditions, running speed limits ranged between 10 kmph and 100 kmph. The vibration signals to be analyzed were obtained as per the following specifications:

- Sampling Frequency: The lowest and highest tire rotation speeds used to collect data were 10 kmph and 100 kmph, respectively. For the 165/80 radial pneumatic air-filled tire examined in this study, 30.1 cm was observed to be the tire radius (R). With respect to the current experimental study, it was observed that the maximum frequency of signals obtained from the accelerometer stood at 14.73 Hz. Along similar lines, the minimum frequency was measured to be approximately 1.47 Hz. A sampling rate of 1 kHz was suggested and selected for this investigation since the Nyquist Sampling Theorem states that a frequency rate of more than or equal to 29.46 Hz should be regarded as the minimum sampling rate [14].
- Sample length: Consistency of the available data plays a crucial part in the balancing of the computational load in this study covering four tire conditions. Thus, an effective sample length of 5000 was chosen.

The wheel considered for this experiment was initially balanced by adding 40 g weights to its rim. The pneumatic tire of the wheel was then inflated to the rated pressure value of 31 psi with air. The term “Normal” was used to describe the vibrational signals obtained under this circumstance. Then, 40 psi pressure was induced in the tire, and the obtained vibrational signals were labeled as being in a “High” state. The tire was deflated from 40 psi to 19 psi in order to achieve the “Puncture” condition. Since signals attained at speeds less than 10 kmph did not possess enough amplitude, they were categorized under ‘Idle’. A total of 60 data points for each signal were extracted and converted into radar plots corresponding to each of the 240 signals. Here, 1200 samples altogether were gathered for various balance circumstances, while the frequency of sampling for each sample with 5000 data points was taken at 1 kHz. Figure 1 presents an overview of the experimental workflow from vibration signal measurement to fault classification.

The tire condition data were obtained from a data acquisition system (DAQ) in the form of comma separated variable (CSV) files comprising amplitude–time data in numeric form. The numeric data in each instance of the target classes were subsequently visualized into radar graphs through the execution of a macro on Microsoft Excel over Visual Basic. The radar plots were then natively stored and resized either into 224 × 224 or 227 × 227 pixels using a resizing algorithm on MATLAB based on the input requirements of each of the pretrained networks under study. These resized images were then fed as input to the pretrained networks for training and classification.

## 3. Convolutional Neural Networks as a Deep Learning Strategy

**Figure 4:**
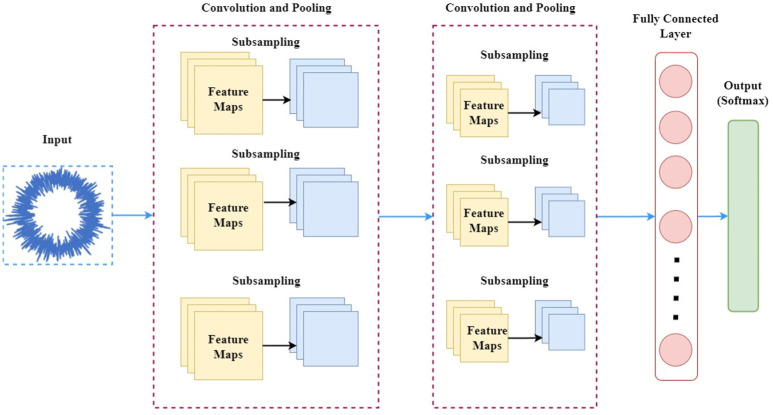
General CNN Architecture and layers involved.

Convolution neural networks are a deep learning approach that function on an automatic feature learning algorithm, which establishes a connection between fed images and features in each of these images based on the biases and weights created and constituted by the CNN. In feature learning and recognition processing of a CNN comprising multiple hidden layers, weights are a measure of signal strength passed between layers of the CNN, while biases are constants that offset passed signal values through arbitrary amounts before passing onto the next layer for computational progress. Ultimately, it is the nature and discreetness of the learned features during convolutions that define the classification ability of the pretrained network advocating the CNN approach for purposes of image classification. From the schematic provided in Figure 4, it is evident that a general CNN architecture generally comprises convolution, pooling and fully connected layers, which are sequentially stacked one after the other. The functionality of a CNN lies in the specific actions that occur in each of the constituting layers, as mentioned below:

1. Image pixel values representing the size of the image are stored by an input layer, which then feeds the same into the convolution layer.
2. The convolution layer is constituted by various weights and biases among the neurons stacked next to the input layer of the CNN. To sustain nonlinearity in the problem, we decided to use rectified linear units (ReLU) as activation functions.
3. The pooling layer or down-sampling layer is the layer next to the convolution layer. To achieve spatial dimensionality and decrease computing complexity, features of higher dimensions are sampled down.
4. The CNN architecture is completed by fully connected layers that yield the results of classification according to the problem being solved. Extracted image features in the data matrix form are flattened. Vectors are obtained from these matrices using the fully connected layers of the CNN.

In effect, CNN transforms the initial input fed into the input layer and quantifies it into class scores that assist the purposes of regression and general classification through downsampling and convolution. This reinforces the fact that determining the overall CNN architecture might not be sufficient to understand the complete functionality of this subset of deep learning. A description of the layers and layer-interconnectivity constituting a general CNN architecture is provided below in Figure 4.

### 3.1. Convolutional Layer

In any network adopting the CNN approach, the process of learning is actuated by the convolution layer. Parameters assigned to layers determine the effective state of several learnable kernels, also called filters, which spread across a wide input range with lower spatial dimensionality at the same time. For each picture the filter receives, activation maps of two dimensions are created during convolution. Every data point that makes up an input picture and goes through the kernel in the convolution layer is thereby converted into the scalar product of weights and volume. The pretrained network being considered is thereafter triggered to acquire available significant features in space through values created by each filter. The weighted sum of the kernel and that of neighboring pixels are calculated and replaced through the input vector over which the midpoint of the kernel is positioned. The complexity of computation can be further reduced by convolution layers using an optimized choice of hyperparameters. Three hyperparameters have a direct and tangible influence on the performance optimization of convolution layers. These include:

- Depth—the total number of filters in the convolution layer.
- Stride—the movement of filters in a particular direction in the convolution layer.
- Zero-Padding—the addition or ‘padding’ using zeros around an input image border.

### 3.2. Pooling Layer

In deep learning approaches, especially in the context of CNN applications, dimensionality reduction of data can be achieved as a specific objective of the pooling layer to eventually reduce the overall computational complexity. This is carried out by shrinking the effective number of computational parameters by acting on every value constituting the input activation map. This layer realizes the dimensionality scaling process through the “MAX” functionality. Depending on their functionality, pooling layers can be classified as “MAX” or “AVERAGE” pooling, with “MAX” pooling being widely adopted as a pooling methodology due to its efficient performance on different types of data. The common setting for the filter size and stride length is 22, which permits pooling layer growth throughout the whole input spatial dimensions.

### 3.3. Fully Connected Layer

Fully connected layers constitute the ultimate computational layers of convolutional neural networks. As the outputs of convolution and pooling layers of CNN (which serve as inputs to the fully connected layer) are generally in matrix form, they must be flattened before being included in the fully connected layer. For this purpose, fully connected activation functions such as sigmoid or softmax are adopted to carry out classification for the given input data. This architecture is an effective approach that augments traditional fault diagnosis methodologies by automating originally redundant intermittent processes in the process of experimentation and subsequent result estimation, owing to the classification and feature selection potential of DNN.

## 4. Experimental Preprocessing and Pre-Trained Network Analysis

**Figure 5:**
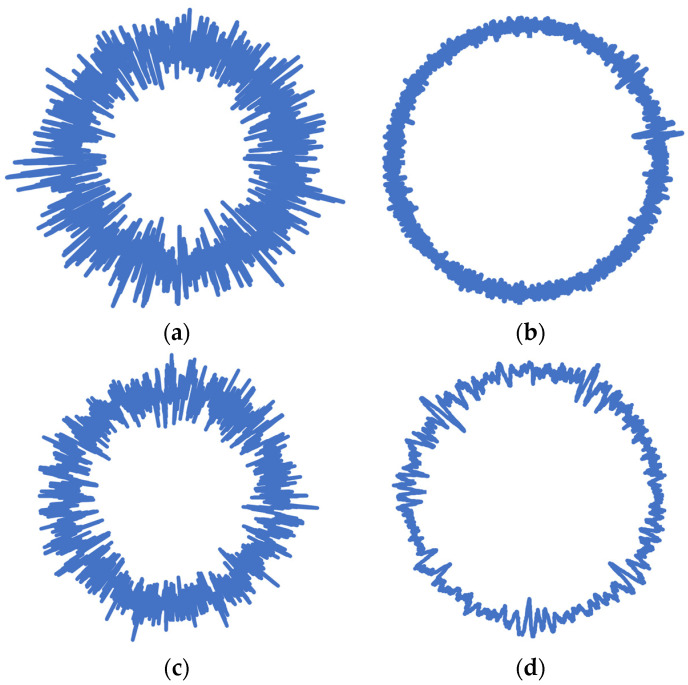
Sample radar plots representing each of the four air-filled pneumatic tire condition cases: (**a**) high, (**b**) idle, (**c**) normal and (**d**) puncture.

Vibration signals acquired for the four different tire conditions of the pneumatic tire equipped on the test vehicle were stored in the form of radar plots as images. A radar plot corresponding to vibration signals measured under normal tire conditions is presented in Figure 5. This was followed by resizing and preprocessing the acquired images into size batches of either 224 × 224 pixels or 227 × 227 pixels. In this research, the networks trained on ImageNet’s initial weights were restored through the transfer learning approach. At the same time, to deploy these pretrained networks to handle a user-customized data set, additional layers in line with the number of user-defined classes replaced the pre-existing output layers. A comparative analysis of the structural makeup of the four different pretrained networks employed in this study is presented in Table 2.

### 4.1. Data Set Constitution and Data Preprocessing

In the current scope of the study, data sets of images comprising tire conditions were made from the vibration signals captured. The four test circumstances, idle, high, puncture and normal, comprised 240 images (with 60 images per class—Idle, High, Puncture and Normal)—essentially, vibrational plots in radial graph form created based on the acquired vibration signal data. These plots/images were subsequently resized to a pixel size of either 224 × 224 or 227 × 227 as per the acceptable configurations for the pretrained network adopted for the computation process in this experiment.

### 4.2. AlexNet Pretrained Network

The AlexNet network, introduced by Alex Krizhevesky in the annual ImageNet Large Scale Visual Recognition Challenge (ILSVRC), comprises 61 million learnable parameters and eight layers, over a preset 1.2 million images with 1000 image classes. Images of size 227 × 227 are accepted as legal entries by this network where the first convolution layer receives input (contains 96 different 11 × 11-pixel filters) that attempt to normalize, max pool and convolve it into 55 × 55 pixels. The second convolution layer accepts the output of the first convolution layer. This second layer is marked by 256 receptive filters, then a layer with a maximum pool size of 3 × 3 pixels. It might be useful to note that rectified linear units (ReLU) are utilized as the roles of activation in each layer to address non-linear conditions. Subsequently, the pixel size of the output image is reduced to 27 × 27 pixels and, upon passing through the third, fourth and fifth convolution layers, is further reduced to a size of 13 × 13 pixels. To ensure that model overfitting does not affect subsequent computations and classification results, a 0.5 ratio dropout layer is augmented prior to a fully connected layer. It is critical to convert spatially intensive matrices into flattened vectorial forms, which are carried out by two fully connected layers stacked on one another, with 4096 learnable parameters in all. Finally, the classification for the considered problem statement is eventuated through means of an output layer with a softmax activation function in the architecture.

### 4.3. VGG-16—Visual Geometry Group-16 Network

In the annual ILSVRC 2014, Karen Simonyan and Andrew Zisserman developed VGG-16, also known as Visual Geometry Group-16 or Oxford Net, which was subsequently hailed as the best-performing network. VGG-16 included a classification layer, three fully connected layers, five max-pooling layers, and 13 convolution layers. The convolution layers were arranged in an image-classification-centric pattern. To further understand the functioning of the VGG-16 model, two learnable parameters termed “A”, and “D” of 3 × 3 filter size can be considered. Naturally, the value of stride coupled with the size value of 3 × 3 pixels can collectively and even individually impact the nature of a convolution layer image output. To prevent model overfitting, each convolution layer has a ReLU activation function enhanced by a dropout ratio of 0.5 (added before the fully connected layer). When an image traverses by means of the convolution layer, the filter is forced to relocate the “x” pixel image whenever a mathematical operation is actuated. This occurs such that the convolution operation produces an output image “z”. The functionality of a convolution operation can be mathematically condensed and interpreted through the medium of the following equation:

z = F(Ax + D)

Max pooling assists in the deconstruction and resizing of input images to augment the extraction of high-importance features with optimized memory utilization. On a general note, the initial layers of the VGG-16 architecture are involved in learning simple image features (for example, edges). On the other hand, the learning of more complex image features is reserved for the deeper layers of this network.

### 4.4. GoogLeNet Pretrained Network

The GoogLeNet Pretrained Network architecture was introduced for application in facial recognition, robotics and adversarial training in the 2014 annual ILSVRC by Szegedy et al. GoogLeNet comprises nine inception modules. These modules are connected to four convolutions, five fully connected, three average pooling, three softmax layers and four max pooling. ReLU is used in fully connected layers as the activation function and is supplemented by a dropout layer with a ratio of 0.5. The solution of more complex computer vision problems by GoogLeNet is enabled through Inception modules present in its architecture, generally by altering the size of the convolution layer filter. This is a major benefit of this network in general and inception modules, in particular, as this assists in a drastic reduction in dimensional complexity and computational time. The drawback is that although GoogLeNet comprises 22 layers and thereby makes it look robust, in practice, the volume of trainable parameters is relatively lesser, especially when compared with the structure of the AlexNet pretrained network.

### 4.5. ResNet-50—Residual Pretrained Network

Developed by He et al., Residual Network (ResNet) was found to be the maximum efficient and successful neural network in the annual ILSVRC 2015. ResNet-50 featured the benefits of a high convergence rate and more accurate classifications. This network was trained on 224 × 224-pixel size color images and applied identity shortcuts characterized by the output identity values being mimicked by the input identity values. The ResNet architecture was constituted by the stacking of residual units. Considering the quantity of residual units and layers present, ResNet designs are available in different forms. The ResNet-50 architecture deployed for this experimental study encompasses a fully connected layer and 49 layers of convolution. While the ResNet architecture also comprises fully connected layers and convolution pooling—as is the case with other pretrained networks discussed and, in fact, also resembles the VGG network architecture—it is found that the ResNet architecture at large is eight times deeper than that of VGG-16, the result of which is a greater number of learnable features and thence, a greater probability for higher degrees of classification accuracy.

## 5. Results and Discussion

The main objective of the present section is to evaluate and comparatively surmise the performance of four pretrained networks for the condition monitoring of an automotive-grade pneumatic air-filled tire, namely, VGG-16, GoogLeNet, AlexNet and ResNet-50. Variations in the training–testing data split ratio, optimizer algorithm, initial learning rate and batch size were carried out using the personal computer version of MATLAB R2020d with the aid of computer vision, transfer learning packages and deep learning toolbox corresponding to each pretrained network. A detailed description of the experimental observations is presented below.

### 5.1. Impact of the Test-Train Ratio

The training–testing data split ratio (or train–test split ratio) is the proportion in which the input data is split into testing and training data sets. The training data set is employed for training the pretrained network by adding to its pre-existent learning, while testing and evaluation are conducted using the testing data set of this trained network. For each of the pre-trained networks, five various training–testing data split ratios were tested by adjusting the additional hyperparameters, including batch size value (10), the solver method (SGDM) and initial learning rate value (0.0001). This assists in the identification of the most optimal train-test split ratio for a given pretrained network being deployed through uniform evaluation. From Table 3, the dependence of the classification performance on the value of the train–test split ratio is evident. Statistically, we observed that AlexNet (Table 3) produced a maximum classification accuracy of 86.10% for a training–testing data split ratio of 0.70:0.30. VGG-16 and GoogLeNet rendered 91.70% and 94.40% accuracy for 0.80:0.20 and 0.85:0.15 train–test split ratios, respectively. The ResNet-50 pretrained network produced a maximum of 89.60% accuracy for the 0.80:0.20 training–testing data split ratio. Thus, the overall classification accuracies of AlexNet, VGG-16, GoogLeNet and ResNet-50 stood at 82.84%, 85.96%, 77.98% and 85.20%, respectively, with the maximum overall accuracy exhibited by the VGG-16 network. It could, therefore, be inferred that the VGG-16 network produced a higher overall classification accuracy of 85.96% with a maximum accuracy of 91.70% for the 0.80:0.20 train–test split.

### 5.2. Effect of Solvers

Solvers (also referred to as optimizers) are algorithms adopted to minimize the training loss value for model performance improvement during the training process. For the purposes of this study, three main solvers, namely, the root mean square propagation method (RMSprop), adaptive moment estimation method (ADAM) and stochastic gradient descent method (SGDM), were individually adopted to evaluate the performance of each model. With respect to the results of Section 5.1, for each of the four pre-trained networks, the optimum train–test split ratios yielding individually higher classification accuracies were as follows:

1. AlexNet—a split ratio between training and testing of 0.70:0.30;
2. VGG-16—a split ratio between training and testing of 0.80:0.20;
3. GoogLeNet—a split ratio between training and testing of 0.85:0.15;
4. ResNet-50—a split ratio between training and testing of 0.80:0.20.

The effectiveness of the pretrained networks may be affected by adjusting the optimizers, and the change of optimizers adopted, as suggested by Table 4. It is evident that the highest classification accuracy in this comparison was obtained by the ResNet-50 network at 93.80% for the RMSprop solver, also amounting to the highest overall classification accuracy of 82.93%. The VGG-16 pretrained network rendered the lowest classification accuracy of 75% for the ADAM solver (which, in its domain, yielded the higher accuracy when compared with the results post-adoption of SGDM and RMSprop) and subsequently the least overall classification accuracy of 63.10%, suggesting that VGG-16 was the worst performing network in this comparison.

### 5.3. Effect of Learning Rate

During every instance that involves an update of the model weight, examining and monitoring the alterations to the training model in accordance with a predictable error is a crucial field to analyze. This can prove to be a challenge as the selection of an ideal learning rate is not directly feasible at face value. This is because a lower value for the learning rate will severely increase the time for computation, while a greater value of initial learning rate might result in a higher error and, therefore, improper training of the model. In this experiment, three initial learning rate values of 0.0001, 0.0003 and 0.001 were set to examine the classification performance of each network, keeping various hyperparameters, including solver and train–test ratio, fixed along the following lines.

1. AlexNet—SGDM solver and 0.70:0.20 train-test split ratio;
2. VGG-16—ADAM solver and 0.80:0.20 train-test split ratio;
3. ResNet 50—RMSprop solver and 0.80:0.20 train-test split ratio;
4. GoogLeNet—SGDM solver and 0.85:0.15 train-test split ratio.

Every pretrained network performs differently depending on the learning rate. This is evident from Table 5. The model has likely learned the features well if the classification accuracy is higher, indicating that the error value is lower. It is, therefore, suggestive that for the hyperparameter choices made so far, the highest classification accuracy was exhibited by ResNet-50 for a learning rate of 0.0001 at 93.80%, rendering the maximum overall accuracy in this comparison at 86.83%.

### 5.4. Impact of Batch Size

The batch size indicates the number of input samples that percolate in batches into a training work during the model training process. This occurs just before the model weight upgradation is carried out. In the current study, five batch sizes—8, 10, 16, 24 and 32—were each coupled with the most optimal choice of hyperparameters obtained so far through the experimental outcomes discussed in earlier sections by fixing the train–test ratio, optimizer and learning rate values. This has been depicted as follows:

1. AlexNet—0.70:0.20 train–test split ratio, SGDM solver, initial learning rate of 0.001;
2. VGG-16—0.80:0.20 train–test split ratio, ADAM solver, initial learning rate of 0.0003;
3. GoogLeNet—0.85:0.15 train–test split ratio, SGDM solver, initial learning rate of 0.0001;
4. ResNet-50—0.80:0.20 train–test split ratio, RMSprop solver, initial learning rate of 0.0001.

From Table 6, it can be observed that the selection of mini-batch size of 10 rendered higher classification accuracies for the VGG-16, GoogLeNet and ResNet-50 pretrained networks, while a batch size of 32 favored higher classification accuracy for the AlexNet network. Specifically, the highest overall accuracy was exhibited by ResNet-50 at 85.44%, with a maximum accuracy of 93.80% observed for a mini-batch size of 10. The setting of an optimal batch size is accompanied by a trade-off between training progress, training time and the generalization capacity of the pretrained network under study. The larger the batch size, the lower the training time, which naturally eventuates into an accelerated and expedited training progress. This is unsafe in the sense that this acceleration in training progress eventuates at the cost of the capability of the network to generalize, which decreases as the values of batch sizes are increased. In summary, an optimal batch size of 10 is suggested for the experimentation of the ResNet-50, GoogLeNet and VGG-16 pretrained networks, and a batch size of 32 is suggested for the analysis of the performance of the AlexNet pretrained model.

### 5.5. Comparative Examination of Trained Models

The overall effectiveness of the pretrained networks is covered in this section, and the choice of optimal hyperparameters that yield maximum classification accuracy is presented. Table 7 prescribes optimal hyperparameter settings that assist the improved effectiveness of pretrained models utilized in this experimental research. Table 8 presents a comparative analysis of the overall performance of pretrained networks with the optimal hyperparameter configurations. In summary, it can be inferred from Table 8 that VGG-16 exhibited the highest performance for its setting of optimal hyperparameters. It is therefore suggested that ResNet-50 be adopted and deployed for tire condition monitoring in automotive applications, owing to its superior classification accuracy and lower computational complexity.

**Figure 6:**
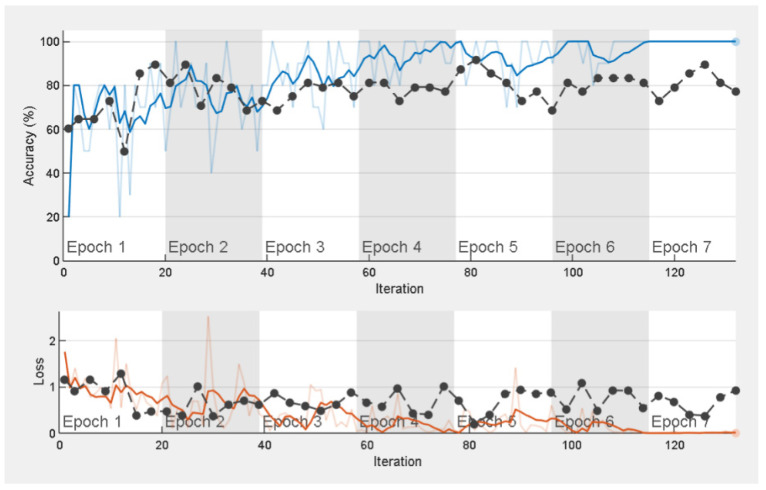
Training progress of ResNet-50 pretrained network.

The training progress of the ResNet-50 network is shown in Figure 6. As the training progress curve saturates by flattening after the sixth epoch, it could be interpreted that the ResNet-50 pretrained network adopted for this study has been trained effectively, and model weights have been successfully upgraded. Additionally, the overall reduction of data losses during the process of network training (in general, for all networks under study) suggests that the optimal choices of hyperparameters have been obtained.

**Figure 7:**
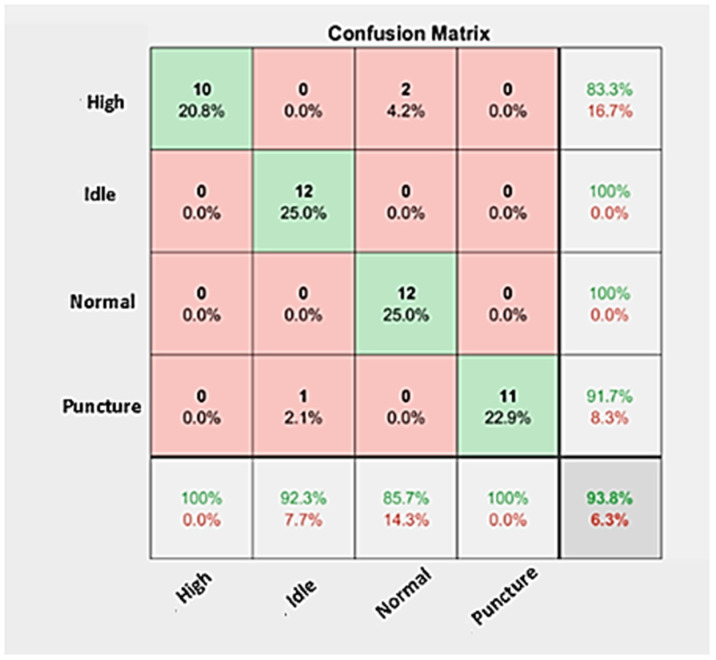
Confusion matrix of the ResNet-50 network.

The confusion matrix corresponding to the classification result for the ResNet-50 architecture deployed in tire condition monitoring is presented in Figure 7. A confusion matrix presents a graphically intuitive approach to interpreting the pretrained network classification performance level. This is evident through the principal diagonal elements of the matrix, which represent correctly classified instances, while incorrectly classified instances (also referred to as misclassified instances) are represented by the non-principal diagonal matrix elements. The absence of misclassified instances infers that the network presents effective transfer learning by the adopter network and minimizes data loss throughout the learning process. The confusion matrix in Figure 7 shows that the ResNet-50 architecture yielded a classification accuracy of 93.80% with no misclassification instances except for Normal (two instances were misclassified as High) and Idle (one instance was misclassified as Puncture), which could have instantiated due to poor signal quality, interruption by noise and discrepancy in similarities between the acquired signals. In summary, Table 8 surmises the performances of optimized pretrained models comparatively and suggests that ResNet-50 can serve as the best-performing and adoptable deep learning pretrained network for the purposes of tire condition monitoring.

### 5.6. Comparison with State of the Art Techniques

To assess the superiority in the performance of the proposed TCMS system fault diagnosis, several state of the art techniques present in the literature were compared. Table 9 presents the overall performance of various state of the art techniques proposed in the literature in comparison to the proposed method. From Table 9, one can observe that the proposed method to diagnose faults in TCMS systems had a maximum classification accuracy of 93.80%.

## 6. Conclusions

Four pretrained deep learning networks, GoogLeNet, VGG-16, AlexNet and ResNet-50, were used to determine the tire condition in this study. Three primary tire conditions—high, low and puncture—and one normal reference state were examined in the research. In this experiment, it was evident that an end-to-end machine learning approach was formulated as these pretrained networks featured layers of CNN and performed feature extraction, feature selection and classification in an integrated approach. Vibration radar graphs were analyzed to deliver classification results based on the hyperparameter configuration of each network. This study serves two purposes in the sense that it not only establishes the fact with evidence that a transfer learning approach for integrated feature selection, extraction and classification could be advocated to execute the regular task of vehicle tire condition monitoring, but also that this approach bears respectable accuracy in classification which, when coupled with ease of experimentation, could serve as a viable commercial alternative to tire condition monitoring. This can be established statistically as the attained experimental outcomes suggest that the considered networks are indeed well accomplished in not just learning complex features but also establishing acceptable classifying results for the purposes of tire condition monitoring. Evidently, hyperparameters such as optimizer, the train–test split ratio, initial learning rate and mini-batch size values were strategically varied, and the best hyperparameters were found for all the networks. As a result, it was observed that ResNet-50 stood as the most optimally performing network with 93.80% accuracy when compared with AlexNet, which produced a classification accuracy of 87.50%, GoogLeNet and ResNet-50, both of which yielded 91.70% as their respective classification accuracies. Among the other networks taken into consideration in the study, ResNet-50 is chosen as the network with the greatest performance, and therefore, ResNet-50 is recommended for performing tire condition monitoring economically. The execution of a program macro to generate radar plots for a single class took 50 s for 60 different signals. Thus, 0.833 s were elapsed for the visualization of each signal. The resizing procedure extended over 2 s, and the generation of a confusion matrix after trained network execution took just under 5 s. Thus, a driver could be alerted about a fault in as low as 6–7 s. The training time for the ResNet-50 network elapsed after 1 min and 30 s (90 s). The future scope of this experiment could include an additional detailed examination of where the accelerometer is located and the performance of said accelerometer in accurate condition classification to ensure higher degrees of classification accuracy through optimal hardware–software synergy. Applications of this conclusion can find their way into commercially available automobiles in the form of modular and user-friendly fault diagnosis and monitoring systems for tire condition monitoring for the purposes of real-time on-the-fly onboard diagnosis.

## Figures and Tables

**Table 1:** Implementation reference of deep learning methods in mechanical applications.

Reference	Deep Learning Approach	Application in Mechanical System
[7]	Convolution Neural Networks with Wavelet Transform	Motor Bearing
[8]	Hierarchical Convolution Neural Network	Roller Bearing
[9]	Convolution Neural Networks with Wavelet Transform	
[10]	Deep Belief Network and Spare Autoencoder	
[11]	Recurrent Neural Network	
[12]	Stacked Autoencoder	Gear Box
[13]	Generative Adversarial Network	
[14]	Convolution Neural Networks	Centrifugal Pump

**Table 2:** Characteristics of networks (pretrained) that implement transfer learning.

Network Name	Number of Layers	Learnable Parameters (in Millions)	Image Input Size (in Pixels)
**VGG-16**	16	137.00	224 × 224
**GoogLeNet**	22	7.10	224 × 224
**AlexNet**	8	60.00	227 × 227
**ResNet-50**	50	25.70	224 × 224

**Table 3:** Pretrained networks performance summary for different training: testing data split ratios.

Pretrained Model	Classification Accuracy for Different Training: Test Data Ratios (%)					Overall Accuracy (%)
	0.60:0.40	0.70:0.30	0.75:0.25	0.80:0.20	0.85:0.15	
**VGG-16**	85.40	87.50	81.90	**91.70**	83.30	**85.96**
**GoogLeNet**	83.30	76.10	76.30	62.50	**91.70**	77.98
**AlexNet**	84.40	**86.10**	83.30	77.10	83.30	82.84
**ResNet-50**	84.40	84.70	86.70	**89.60**	80.60	85.20

**Table 4:** Performance of pretrained networks for different solvers.

Pretrained Model	Classification Accuracy for Different Solvers (%)			Overall Accuracy (%)
	SGDM	ADAM	RMSPROP	
**VGG-16**	70.80	**75.00**	43.50	63.10
**GoogLeNet**	**86.10**	75.00	83.30	81.47
**AlexNet**	**86.10**	72.20	70.10	76.13
**ResNet-50**	69.60	85.40	**93.80**	**82.93**

**Table 5:** Performance variation of pretrained networks with change in initial learning rates.

Pretrained Model	Accuracy of Classification for Various Learning Rates (%)			Overall Accuracy (%)
	0.0001	0.0003	0.001	
**VGG-16**	72.90	**79.20**	52.10	68.07
**GoogLeNet**	50.60	86.00	**86.10**	74.23
**AlexNet**	72.20	83.20	**83.30**	79.57
**ResNet-50**	**93.80**	77.10	89.60	**86.83**

**Table 6:** Performance of pretrained networks for different minibatch sizes.

Pretrained Model	Accuracy of Classification for Various Mini-Batch Sizes (%)					Overall Accuracy (%)
	8	10	16	24	32	
**VGG-16**	66.70	**91.70**	25.00	70.80	68.80	64.60
**GoogLeNet**	88.90	**91.70**	69.44	80.60	86.10	83.35
**AlexNet**	84.70	83.30	81.90	81.90	**87.50**	83.86
**ResNet-50**	79.20	**93.80**	91.70	75.00	87.50	85.44

**Table 7:** Optimal hyperparameters for pretrained model.

Pretrained Network	Hyperparameter Configuration				Overall Accuracy (%)
	Train–Test Split Ratio	Optimizer/Solver Algorithm	Initial Learning Rate	Batch Size	
**VGG-16**	0.80:0.20	ADAM	0.0003	10	91.70
**GoogLeNet**	0.85:0.15	SGDM	0.0001	10	91.70
**AlexNet**	0.70:0.30	SGDM	0.001	32	87.50
**ResNet-50**	0.80:0.20	RMSPROP	0.0001	10	**93.80**

**Table 8:** Performance comparison of pretrained models with optimal hyperparameters.

Pretrained Network	VGG-16	GoogLeNet	AlexNet	ResNet-50
**Classification Accuracy (%)**	91.70	91.70	87.50	**93.80**

**Table 9:** Comparison of tire condition monitoring approaches with our proposed method.

Reference	Tire Condition Monitoring Approach	Sensor Used	Classification Accuracy(%)
[29]	Statistical Feature Extraction and K-Star Algorithm	MEMS accelerometer	89.16
[30]	Statistical Features and Support Vector Machine Algorithm	MEMS accelerometer	90.00
[31]	Statistical Analysis and Regression Algorithm	MEMS accelerometer	91.25
[32]	Statistical Analysis and Logistic Model Tree	MEMS accelerometer	92.50
[33]	Statistical Analysis and Rotation Forest Algorithm	MEMS accelerometer	93.33
	Proposed Method—Radar Plots and Pretrained Neural Networks	MEMS accelerometer	**93.80**

## Data Availability

Not applicable.
